# A pilot study on essential oil aroma stimulation for enhancing slow-wave EEG in sleeping brain

**DOI:** 10.1038/s41598-020-80171-x

**Published:** 2021-01-13

**Authors:** Li-Wei Ko, Cheng-Hua Su, Meng-Hsun Yang, Shen-Yi Liu, Tung-Ping Su

**Affiliations:** 1 Center for Intelligent Drug Systems and Smart Bio-Devices (IDS2B), National Chiao Tung University, Hsinchu City, Taiwan; 2 Institute of Bioinformatics and Systems Biology, National Chiao Tung University, Hsinchu City, Taiwan; 3 Drug Development and Value Creation Research Center, Kaohsiung Medical University, Kaohsiung City, Taiwan; 4 Sleep Center, Taipei Veterans General Hospital, Taipei City, Taiwan; 5 Department of Psychiatry, Cheng Hsin General Hospital, Taipei City, Taiwan; 6 Department of Psychiatry, Faculty of Medicine, National Yang-Ming University, Taipei, Taiwan

**Keywords:** Neuroscience, Neurology

## Abstract

Sleep quality is important to health and life quality. Lack of sleep can lead to a variety of health issues and reduce in daytime function. Recent study by Fultz et al. also indicated that sleep is crucial to brain metabolism. Delta power in sleep EEG often indicates good sleep quality while alpha power usually indicates sleep interruptions and poor sleep quality. Essential oil has been speculated to improve sleep quality. Previous studies also suggest essential oil aroma may affect human brain activity when applied awake. However, those studies were often not blinded, which makes the effectiveness and mechanism of aroma a heavily debated topic. In this study, we aim to explore the effect of essential oil aroma on human sleep quality and sleep EEG in a single-blinded setup. The aroma was released when the participants are asleep, which kept the influence of psychological expectation to the minimum. We recruited nine young, healthy participants with regular lifestyle and no sleep problem. All participants reported better sleep quality and more daytime vigorous after exposing to lavender aroma in sleep. We also observed that upon lavender aroma releases, alpha wave in wake stage was reduced while delta wave in slow-wave sleep (SWS) was increased. Lastly, we found that lavender oil promote occurrence of SWS. Overall, our study results show that essential oil aroma can be used to promote both subjective and objective sleep quality in healthy human subjects. This makes aroma intervention a potential solution for poor sleep quality and insomnia.

## Introduction

Sleep is one of the most fundamental physical requirements for human survival, and increasingly viewed as playing an important role in restitution of human body^1^. An important aspect of well-being, sleep quality is closely related to overall quality of life, secretion of the stress hormone, cortisol, and immunity^2^. On the other hand, sleep deprivation is prevalent in modern society. Lack of sleep is known to contribute to a wide range of physical and mental health issues including impaired immunity^3^, memory loss^4^, obesity^5,6^, increased risk of cardiovascular disorder^7^, and more. Although sleep quality can be improved by hypnotics, like benzodiazepines and zolpidem, these medications can lead to other issues including sleep walking^8^, memory loss^9^, and impaired cognitive function^10^.

It’s long speculated that essential oils from some plants may help improve sleep quality, either through inhaling or applying on skin. One of the most commonly used essential oil is lavender oil, which has been found to improve sleep quality and mood^11–15^. However, the mechanism behind various oil’s effect on human brain was not well explored. Therapies and interventions involving aroma has not been accepted by mainstream science and are often considered pseudoscience. One of the issues withholding research on aroma is that designing a blinded experiment for aroma inhalation is hard. It’s difficult to find an innocuous placebo that shares a similar smell with the aroma of interest^16^. As such, subjects are often not blinded in previous studies. What’s worse, most studies on aroma were heavily focused on behavioral changes, which make their results even more susceptible to participants' perceptions and expectations. While Chien et al.^13^ demonstrated in their study that lavender oil helped reduce insomnia, Howard and Hughes’ study^17^ suggested that psychological expectations may have more effect than the aroma itself. Without further understanding on mechanisms of essential oil aroma, it is hard to evaluate its effectiveness and improve the efficiency of its application. Alternatively, neuroimaging technology like electroencephalography (EEG) could provide a more objective insight into aroma’s effect than behavioral studies. An additional benefit of EEG is that it can be performed when the subject is unconscious. Conducting aroma study in sleep time would exempt the need to find a similar smelling placebo and keep the influence of subject expectancy to minimal. Therefore, EEG could be an effective method in studying effects of aroma intervention on human sleep and human brain in general.

Sleep EEG has an episodic variation that can be divided into different stages by trained technicians^18,19^. According to guideline provided by American Academy of Sleep Medicine (AASM), these stages include wake (W), non-rapid eye movement (NREM) stage 1 (N1), NREM stage 2 (N2), NREM stage 3 (N3), and rapid eye movement (REM)^20^. These stages can be identified through polysomnography (PSG), which is commonly used to record various bio-signals like EEG, heart rate, nasal air flow and body positions during sleep. The wake stage is dominated by alpha wave (8–12 Hz) activity in the brain and indicates brief interruption of sleep, of which the person may or may not be aware. The N1, N2, and REM stage are regarded as light sleep during which brain waves slow down, but the person is still easy to wake. N3 is dominated by slower delta wave (0.5–4 Hz) activity, which gives it the alternative name “slow-wave sleep” (SWS). Frequent occurrences of wake stage and increased alpha wave activity usually indicate poor sleep quality, while increased N3 and delta wave activity reflects good sleep quality^21,22^. Recent study by Fultz et al.^23^ also suggested that SWS is crucial to cerebrospinal fluid (CSF) flow and removal of toxic materials from brain. Previous study done by Goel et al.^11^ found that inhaling lavender aroma before sleep could increase SWS and subjective sleep quality. Other studies also reported that inhaling lavender aroma increases theta (4-7 Hz) wave^24,25^ throughout the whole brain during waking times. These studies suggest that lavender aroma may promote low-frequency (theta & delta) brain waves, which could increase deep sleep and improve individual sleep quality. In this study, we present a single-blinded approach to explore inhaled essential oil aroma’s effect on human sleep. We recruited healthy people as a proof of concept to see how aroma may affect sleep stages, sleeping brain activity, and overall sleep quality.

## Methods

### Participants

Nine healthy, right-handed volunteers participated in this study (four females and five males; overall mean age 22 ± 2 years; males: 21.5 ± 0.5 years; females: 22.8 ± 3 years). All participants were asked to keep a regular daily routine and filled out their daily sleep schedules for 1 week before the experiment. All participants gave clear, informed written consent before participating the experiment. None of the participants reported having any smell disorder, relevant history of medications used, smoking, nasal allergies, night shifts or slept during daytime within the previous month. The experiment and methodology were vetted and approved by the Institutional Review Board (IRB) of National Chiao Tung University. All experimental methods follow the guideline of Taiwan Society of Sleep Medicine (TSSM)^26^ and were performed in accordance with local legal regulation.

### Sleep laboratory

The sleep laboratory was designed based on a typical home bedroom to provide a comfortable environment for participants. The sleep laboratory was divided into two parts: an experimental room and a monitoring room. The experimental room had a bed with sheets and pillow. Two infrared cameras were mounted in the experimental room. During the experiment, a trained personnel would stay in the monitoring room to monitor the participant. Both rooms were air-conditioned and the temperature was maintained between 24 to 26 degrees centigrade.

### Questionnaires

This study used various questionnaires to assess parameters related to the participants’ subjective sleep quality. All of these were designed based on Practice of Sleep Medicine^26^, which is the official guideline of TSSM. A pre-session questionnaire (questionnaire A) was administrated before the sleep session started. It includes 11 questions about the participant’s daily activity, their psychological status and their intake of alcoholic/caffeine drink (Table S1). This questionnaire was used to ensure that the participant was in their usual state during monitored sleep sessions. A post-session questionnaire (questionnaire B) was administrated right after the sleep session ended. It includes 12 multiple choices questions about the participant’s sleep experience (Table S2). Like the sleep diary, questionnaire B would derive 4 sleep quality indexes that reflect the participant’s subjective sleep quality. Various Stanford Sleepiness (SSS) questionnaires^27^ were filled in the following day to evaluate daytime sleepiness of the participant on a scale of 1 to 7 (Table S3) (Higher means sleepier).

### Experiment procedure

**Figure 1:**
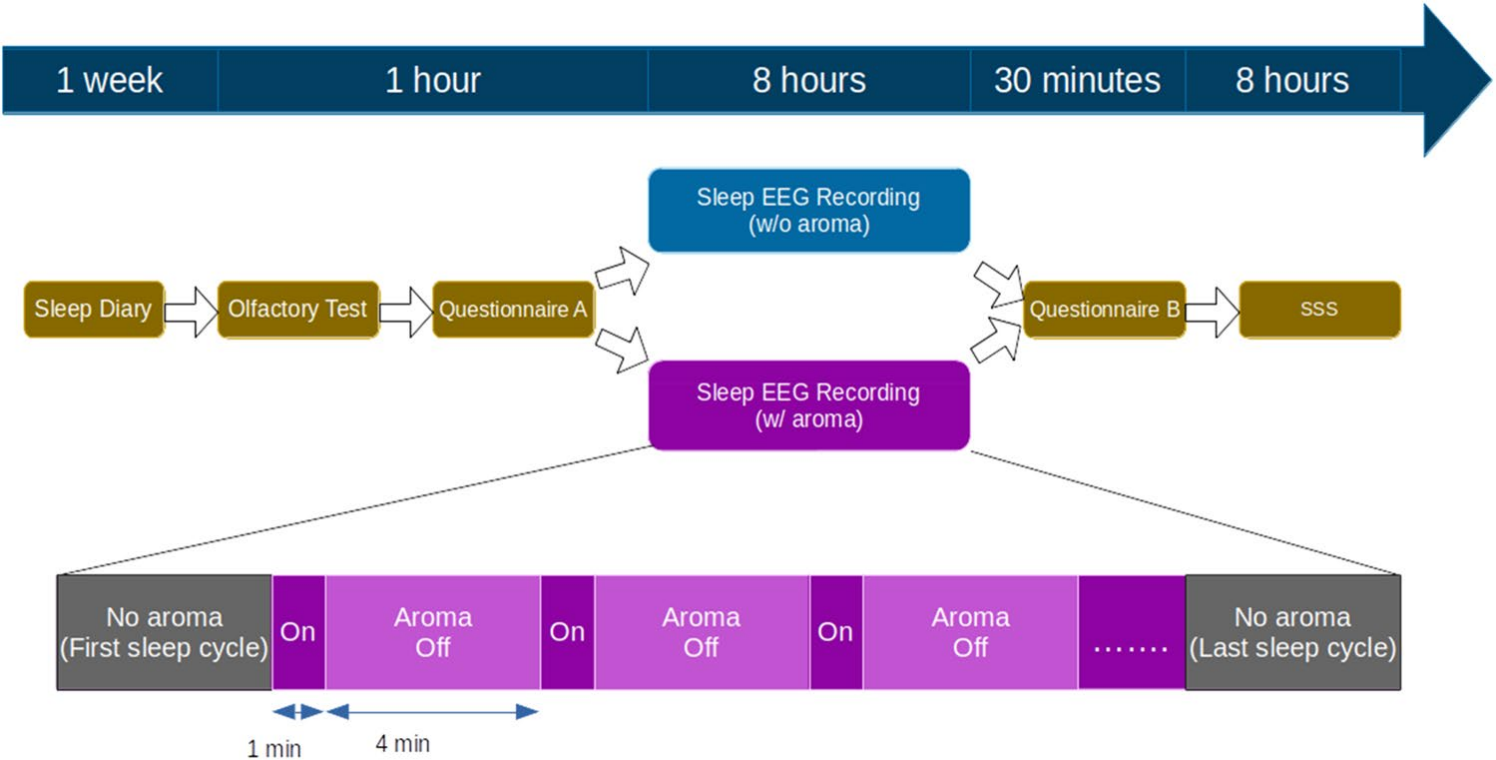
Experimental flowchart.

Each participant was recorded for two nights. One of the nights would be chosen as the stimulus night while the other night was used as control. The orders of control night and stimulus night among the participants were counterbalanced. Half of the participants would receive aroma stimuli at first night while the other half would receive the stimuli on second night. Before both stimulus and control night, the participants were told to do their usual activities and not to take a nap or any alcoholic/caffeine drink during the preceding day. The participants would arrive at the sleep lab at around 10:30 PM to perform an olfactory function check and fill out questionnaire A. They would then go to bed at 11:30 PM and be woken at 7:30 AM. When the participants woke up, they were asked if they noticed any smell during their sleep. They were then asked to fill out questionnaire B. During the ensuing day, the participants were asked to record a SSS questionnaire score every 2 h from 8:00 AM to 4:00 PM (Fig. 1).

During the stimulus night, essential oil of *Lavandula angustifolia* was repeatedly released starting from 1.5 h after bedtime to 1.5 h before the waking. This is done to prevent the participants from noticing the scent of essential oil. Each release was held for 1 min and then dropped for 4 min to avoid olfactory fatigue (Fig. 1). During the control night, the essential oil dispenser was configured as in stimulus night to avoid alerting the participant. Instead of essential oil, water vapor was released in control night.

### Signal recording and processing

EEG, EOG and EMG signals were recorded using the CURRY Scan NuAmps Express system (Compumedics Neuroscan, Charlotte, NC, USA). Electrodes were secured for recording 20 channels of EEG with the 10/20 International Placement System. All EEG signals were re-referenced to the opposite lateral mastoids. Two EOG channels (LEOG, REOG) and one chin EMG were recorded simultaneously to help classifying sleep stage. All signals were recorded at a sample rate of 500 Hz.

Signals from 8 EEG channels (F3, F4, C3, C4, P3, P4, O1, and O2), all EOG channels and the chin EMG channel were used for sleep stage classifying. The signals were filtered with a band-pass filter at 10–70 Hz and then resampled to 128 Hz. All signals were then divided into 30-s epochs. Each epoch was scored and labeled visually based on the manual scoring rules of AASM^20^ by an experienced sleep technologist. All signal processing and analysis were performed using MATLAB (2019a., The MathWorks, Natick, MA, USA).

**Figure 2:**
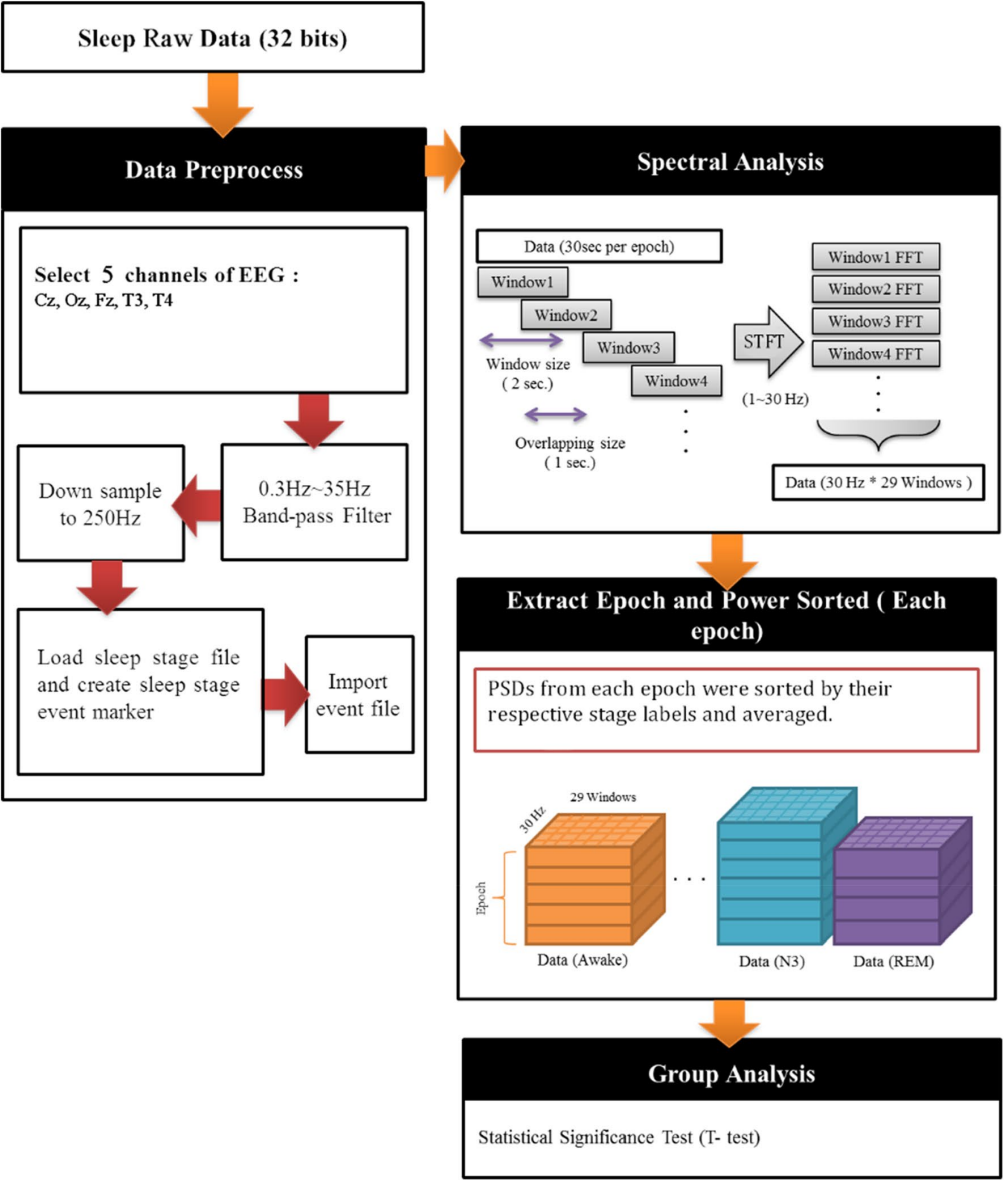
EEG power spectrum density analysis flowchart.

To observe EEG spectral changes induced by lavender stimuli, EEG epochs from control night and stimuli night during the lavender aroma releases were selected to generate power spectrum (Fig. 2). Raw signals from Fz, Cz, and Oz channels were filtered with a band-pass filter at 0.3–35 Hz and a 60 Hz notch filter. They were then resampled from 500 to 250 Hz to reduce computational complexity. We used short-time Fourier transform (STFT) with 2-s Hamming window overlapped with a 1-s window to estimate their power spectrum density (PSD) of the epochs. For each EEG channel, the first two N1 epochs after sleep onset were used as the baseline epochs, as their variations were smaller than that of the first PSG epoch (usually wake stage). The average PSD of the two epochs was used as the baseline PSD for each channel. PSDs from various EEG epochs were grouped by their sleep stages label and then averaged. Power spectra of. Power spectra of T3 and T4 were derived in the same manner to observe olfactory-related activities in temporal lobe.

### Statistical analysis

Subjective (questionnaires) and objective (PSG reports) sleep quality comparisons were done by using Wilcoxon signed-rank test due to small sample size (nine participants) and the results don’t follow normal distribution. As for EEG STFT PSD analysis, the Student t-test is used because the data amount is large enough.

## Results

### Effect on subjective sleep quality

**Figure 3:**
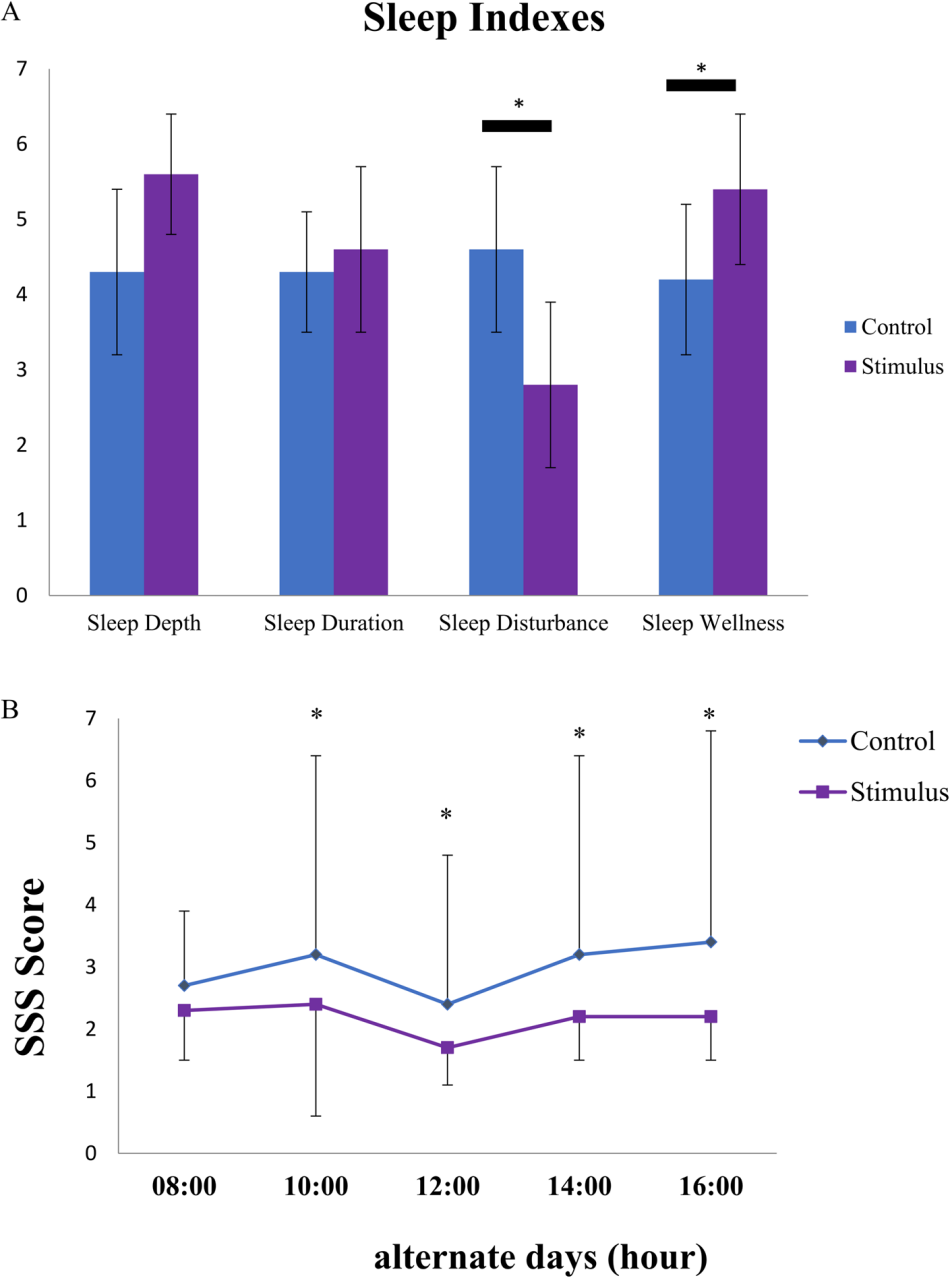
Lavender aroma stimuli improved subjective sleep quality. (**A**) Sleep indexes from questionnaire B. Sleep Disturbance was decreased while sleep wellness was increased in stimuli night. (**B**) Daytime SSS questionnaire ratings. SSS rating after stimuli night was higher than that of control night from 10:00 AM to 4:00 PM.

When being asked, none of the nine participants reported noticing scent of lavender during their sleep session in either stimulus night or control night. Participants showed significant differences in subjective sleep quality scores between post-session questionnaires from the stimulus nights and those from control nights (Fig. 3A). Though ratings of sleep depth and sleep duration were roughly the same between stimulus and control nights (sleep depth: 5.6 ± 0.8 vs. 4.3 ± 1.1; p > 0.5; sleep duration: 4.6 ± 1.1 vs. 4.3 ± 0.8; p > 0.5), the rating of sleep disturbance in stimuli night was lower than in control night (2.8 ± 1.1 vs. 4.6 ± 1.1; p = 0.01). On the other hand, ratings of sleep wellness from stimulus night were higher than control nights (5.4 ± 1.0 vs. 4.2 ± 1.0; p = 0.01). The SSS questionnaire ratings after the stimulus nights were significantly lower than those following the control nights (Fig. 3B). These results show that lavender aroma could improve subjective sleep quality.

### Lavender aroma induced EEG changes in different sleep stages

**Figure 4:**
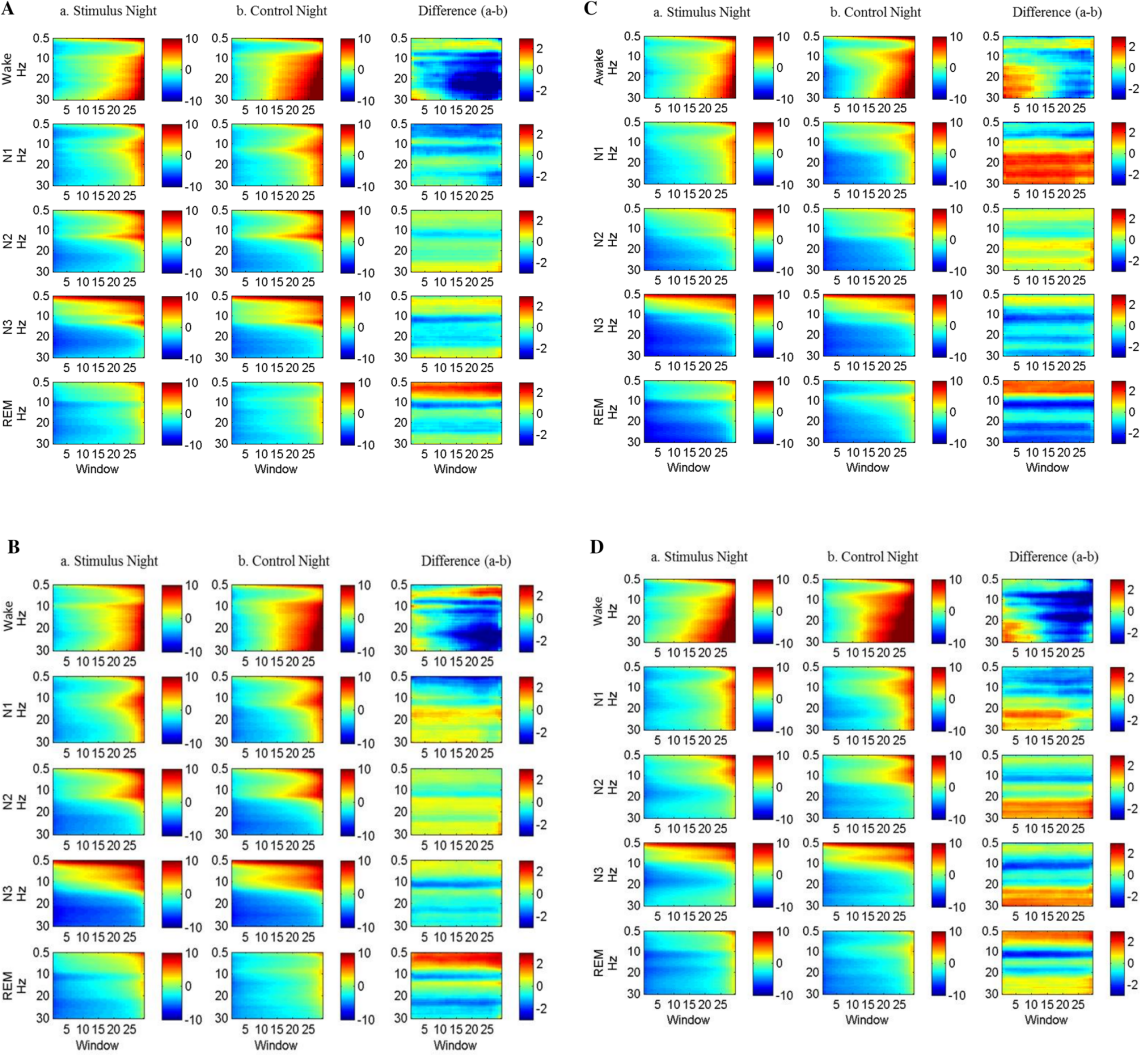

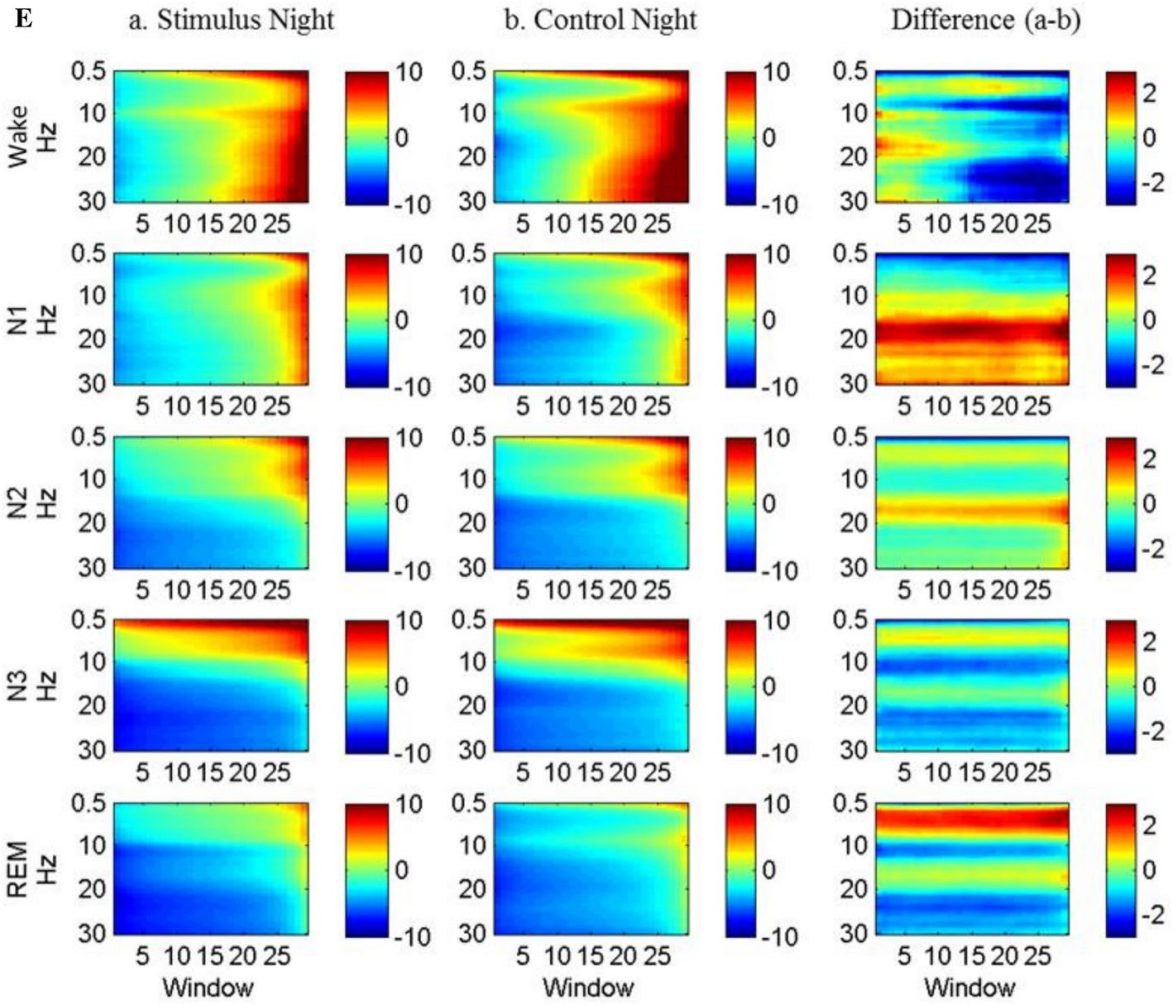
EEG spectrum during the release of lavender oil. (**A**) Cz channel, (**B**) Fz channel, (**C**) Oz channel, (**D**) T3 channel, (**E**) T4 channel.

Since lavender aroma improved subjective sleep quality, we proceeded to inspect its effect on brain wave activity by analyzing the EEG power spectra changes upon release of lavender aroma. The analysis showed that lavender aroma affected multiple brain regions across different sleep stages (Fig. 4). In wake stage, alpha and beta power were significantly decreased in Cz, Fz, Oz, T3, and T4. Additionally, the Fz channel showed increased slow delta activity towards the end of wake stage. The decreased alpha activity across multiple brain regions, especially in the motor cortex and frontal lobes, implies that there were less sleep interruptions and limb movements during the stimulus night.

In N3 stage and REM stage, T3 and T4 (representing left and right temporal lobe respectively) showed a slightly different pattern. While T3 showed increased beta power in N3, T4 showed decreased beta power. All the five channels, including T3 and T4, showed significant decreases in alpha power and increases in delta power. This result indicates that sleep during stimulus nights was deeper and more stable. Similar changes could also be seen during REM stage as well.

### Lavender aroma induced changes in sleep stages

**Figure 5:**
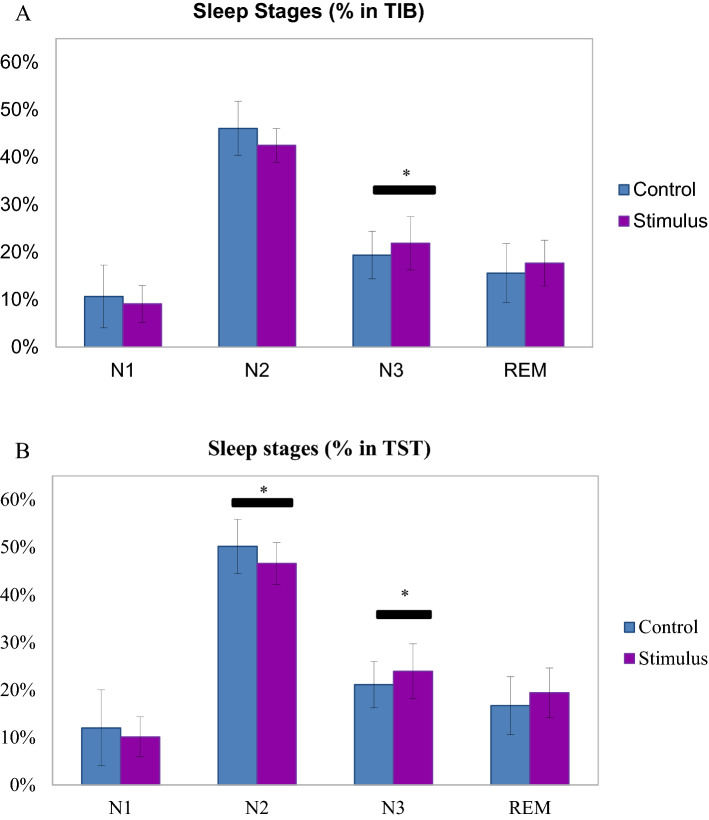
Lavender aroma promotes deeper sleep stages. (**A**) Sleep stages percentage in TIB. Percentage of N3 stage was increased in stimuli night. (**B**) Sleep stages percentage in TST. Percentage of N3 stage was increased while that of N2 was decreased in stimuli night.

**Figure 6:**
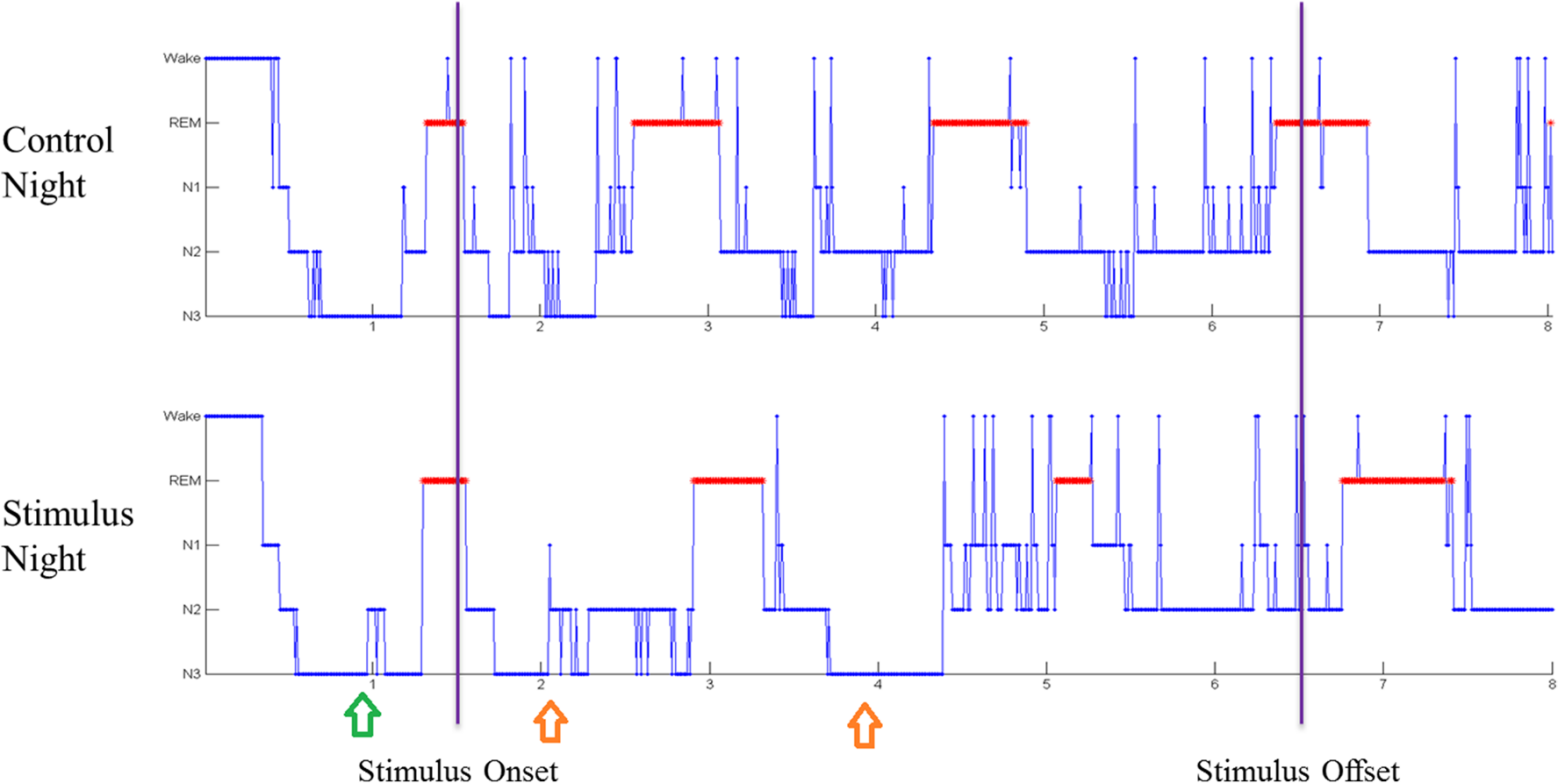
Hypnogram of one of the participants (S1) drawn by sleep technician. N3 showed the same pattern in stimuli night and control night before stimuli onset (green arrow). Upon stimuli onset, N3 became longer and more frequent (orange arrow).

We proceeded to examine the distribution of sleep stages among our participants. The sleep EEG recordings showed that there was no difference in total time in bed (TIB) (455 ± 66 min vs. 453 ± 53 min; p > 0.05) between stimulus and control conditions. There was no difference in sleep efficiency (SE) (91.1 ± 5.9% vs. 91.8 ± 4.8%; p > 0.05), and wake after sleep‐onset (WASO) (8.9 ± 5.9% vs. 8.3 ± 4.8%; p > 0.05) either. These suggest that the participants slept for roughly the same amount of time in both control and stimulus nights (Table 1). While the time percentages of N1, N2 and REM relative to TIB showed no difference between conditions, time percentage of N3 for the stimulus night was significantly higher than that for control night (21.9 ± 5.6% vs. 19.4 ± 5%; p = 0.01) (Fig. 5A). This suggests that although participants slept for roughly the same time with or without lavender aroma, they spent more time in N3 when the lavender stimulus was present. Similar phenomenon can be seen when we replaced TIB with total sleep time (TST). Time percentage of N3 relative to TST is higher for the stimulus night than that for the control night (23.9 ± 5.8% vs. 21.1 ± 4.9%; p = 0.01). In contrast, percentage of N2 was lower for the stimulus night than for the control night (46.6 ± 3.3% vs. 50.2 ± 5.7%; p = 0.05) (Fig. 5B). When examining the participants’ hypnograms, we found that the N3 stages were longer and more frequent upon lavender releases (Fig. 6). These results confirmed that sleep in stimulus nights were indeed deeper and more stable. Overall, these results suggest that lavender aroma can reduce alpha power and promote delta power in the sleeping brain, leading to deeper sleep and improved sleep quality.

**Table 1:** Sleep EEG measures.

	Control night	Stimulus night	p value
TIB (min)	452.9 ± 53.4	455.1 ± 65.7	0.74
TST (min)	403.6 ± 51.6	455.1 ± 65.7	0.41
SE (%)	91.8 ± 4.8	91.1 ± 5.9	0.73
WASO, %TIB	8.3 ± 4.8	8.9 ± 5.9	0.50
WASO, latency (min)	14.45 ± 13.6	13.95 ± 6.4	0.42
WASO, duration (min)	38.1 ± 23.7	40.2 ± 28.9	0.54
N1, duration (min)	49.4 ± 32.9	41 ± 18.6	0.41
N1, %TIB	10.7 ± 6.6	9.1 ± 3.9	0.57
N1, %TST	12 ± 8	10.1 ± 4.2	0.82
N2, duration(min)	208.7 ± 35.8	194.2 ± 36.1	0.42
N2, %TIB	46.1 ± 5.7	42.5 ± 3.5	0.14
N2, %TST	50.2 ± 5.7	46.6 ± 3.3	0.05
N3, duration (min)	85.7 ± 12.9	96.7 ± 16.4	0.05
N3, %TIB	19.4 ± 5	21.9 ± 5.6	0.01
N3, %TST	21.1 ± 4.9	23.9 ± 5.8	0.03
REM, duration (min)	71.1 ± 31.2	83.0 ± 26.1	0.49
REM, %TIB	15.6 ± 6.2	17.7 ± 4.8	0.44
REM, %TST	16.7 ± 6.1	19.4 ± 5.2	0.25

## Discussion

There have been multiple claims about essential oil’s positive effects on sleep quality over the last two decades^11–15^. However, the mechanisms of aroma remain a debated topic. The nature of aromatherapy has made it hard to exclude psychological factors from experiment designs. In this study, we designed a single-blinded experiment to overcame the limitation of aroma study. By randomly selecting stimulus nights and only releasing lavender aroma when the participants were asleep, we were able to keep the participants blinded and reduce influence of their psychological expediencies to the minimum. All participants experienced less disturbance and better sleep quality during stimuli nights. Their EEG recordings further confirmed the benefit of lavender oil as they showed increased delta activity in deep sleep and reduced alpha/beta activity during wake stages. Our study not only suggests that lavender aroma may promote sleep quality by influencing sleep brain activity directly, but also demonstrated that blinded study of aromatherapy is achievable.

In their subjective questionnaires, the participants reported decreased sleep disturbance and increased sleep wellness during stimulus nights. Although total wake stage duration was not reduced during stimulus nights, EEG power analysis shows that wake stage alpha and beta power were reduced in frontal lobe and motor cortex. Previous study done by Halász et al.^28^ suggested that there are different types of awakening in mammal sleep. Schwabedal et al.’s study^22^ found that short WASOs have much less alpha activity than longer (> 5 min), more perceivable WASOs. They went on to propose that the long and short WASOs might be fundamentally different. Although our studies did not observe any difference in WASO duration between control and stimulus night, our participants did report reduced sleep disturbances in stimulus nights. It is possible that by reducing alpha activity, lavender aroma was able to make longer WASOs more like shorter WASOs and make them less perceivable for the participants, though more studies need to be done to verify this phenomenon.

During the experiment, the participants showed no significant differences in SE between stimulus and control nights. This suggests that lavender aroma did not affect the sleep/wake ratio in healthy young adults. On the other hand, the participants showed increased SWS and reduced N2 in stimulus night. The increase of SWS is synchronous with lavender releases as shown in Fig. 6, suggesting that these increases are caused by lavender oil stimuli. SWS is not only important to daytime vigor but also overall health of brain as well. Recent study by Fultz et al.^23^ revealed that occurrence of SWS is followed by large volumes of fluid exchange in human brain. This suggests that SWS is not just vital for daytime vigor and performance, but also removal of harmful materials. Several neurodegenerative disorders like Alzheimer’s disease (AD) and Parkinson’s disease (PD) are related to accumulation of toxic protein debris^29,30^. Study by Varga et al.^31^ has shown that elderly people with longer SWS time have lower concentration of AD-inducing protein debris in CSF when they woke up. Our finding suggests that lavender aroma may be used to increase SWS in sleep and help prevent neurodegenerative diseases even when total sleep time is limited. As sleep time is often limited due to the work constraints in modern society, sleep-time lavender aroma may provide a cheap, safe way to improve sleep quality and prevent diseases like AD with minimal alteration of personal schedules and/or sleep/wake cycle.

We observed that temporal activity is significantly increased upon lavender aroma release. This confirms previous studies suggesting that the human brain can process olfactory stimuli during sleep^32,33^. The increased activities in the temporal lobes were simultaneous with increased delta power and decreased alpha power in other brain regions. This suggests the increased sleep quality is directly related to aroma stimuli. Perl et al.^34^ found that lavender stimuli increase delta activity in NREM sleep. Their results showed that odor release duration is positively related with the time span of increased delta activity, which is consistent with our study. On the other hand, studies on wake time EEG reported that lavender aroma increases alpha and theta wave activity instead of delta activity^24,25^, which are different from our and Perl et al.’s results. This implies that sleeping brain and wake brain react differently to lavender stimuli. More studies are required to clarify how lavender aroma affects brain activity. It is also worth exploring aroma of other essential oils or herbs that are suspected to improve sleep quality, such as chamomile^35^.

This study observed that lavender aroma promotes deep sleep when inhaled asleep. In the stimulus night, SWS percentage was increased and N2 percentage was decreased in relation to TST. It is not clear how lavender aroma achieve such effect, either by pushing brain in N2 stage into SWS, or by keeping brain in SWS from bouncing back to light sleep. More studies need to be done to determine which one is the dominant mechanism. In both cases, lavender aroma could benefit from automated, real-time sleep stage classifying. By releasing the aroma at the right stage through brain-computer interface (BCI), we can maximize the effect of aroma appliance. Curiously, previous study by Rasch et al. showed that odor cue during SWS can promote consolidation of memory related to the cue^32^. This means that a BCI-controlled aroma releasing system may not only improve sleep quality and toxic material removal but also aid in daytime learning. Automated sleep stage classifying is a hot topic in recent years^36–38^, though most proposed methods conduct the classifying post hoc instead of in real-time. We’re currently improving a time-independent classifying algorithm we proposed earlier^39^. The algorithm only requires two forehead EEG channels for feature extraction, which makes it particularly suitable for sleep BCI application. We’re looking forward to exploring the potential of BCI-controlled SWS enhancing system.

Linalool, a compound that can stimulate parasympathetic neurons^35^, has long been suspected to be the key to lavender’s effect in reducing anxiety and improving sleep quality. Oral extract of *Lavandula angustifolia* containing linalool has been reported to improve sleep quality in patients with anxiety disorder^40^. However, recent study done by Seifritz et al.^41^ suggested that the extract has a long (about 2 weeks) delay in its effect on sleep quality and does not improve sleep quality in subjects with low anxiety. In this study, we demonstrated that lavender aroma stimulus changes the brain activity and improve sleep quality very quickly. Our participants reported experiencing better sleep quality and more daytime vigor after just one night of exposure. Our findings hint that inhaling essential oil may influence brain activity through different mechanism than that of oral consumption of lavender or lavender extract. A combination of oral extract intake and sleep-time aromatherapy may achieve greater effects than applying these two methods separately. As insomnia becomes more prevalent in modern society, lavender may provide a safe, effective way in combating sleep-related problems.

Despite showing promising result, this study has some major limitations. The first limitation is that the sample size was rather small. The small sample size may not be strong enough to justify a new aroma-based treatment for sleep deprivation or other sleep disorder. The second limitation is that our study only included young adults. The aim of this study is to explore the effect of aroma oil on human sleep pattern and EEG. More studies with bigger and more diverse sample would be needed to determine the underlying mechanism and clinical potential of essential oil aroma on sleep.

## Conclusion

By utilizing a single-blinded design, our study was able to demonstrate that essential oil aroma may have positive effects on objective and subjective sleep quality in healthy young adults. None of our 9 participants reported smelling lavender during stimulus night, while experience more vigor in the following day. EEG power analysis showed that our participants showed decreased alpha activity and increased delta activity upon aroma release. These results suggest that the improved sleep quality is directly linked to aroma. Furthermore, our study found that SWS was increased in stimulus night. This study could serve as a pilot for future study on aroma and cognitive function and improve application of aroma in the future.

## Supplementary Information

Supplementary Information
