# Supplementary material for: A pilot study on essential oil aroma stimulation for enhancing slow-wave EEG in sleeping brain
Source: Sci Rep. 2021 Jan 13;11:1078. doi: 10.1038/s41598-020-80171-x (PMC7806966; doi:10.1038/s41598-020-80171-x)
Supplement: Supplementary file 1 — Supplementary Information [file 41598_2020_80171_MOESM1_ESM.docx]

**A Pilot Study on Essential Oil Aroma Stimulation for Enhancing Slow-Wave EEG in Sleeping Brain**

Li-Wei Ko^1,2,3,*^, Cheng-Hua Su^1,2^, Meng-Hsun Yang^2^, Shen-Yi Liu^4^, Tung-Ping Su^5,6^

Affiliation

1. Center for Intelligent Drug Systems and Smart Bio-devices (IDS2B), National Chiao Tung University, Hsinchu City, Taiwan
2. Institute of Bioinformatics and Systems Biology, National Chiao Tung University, Hsinchu City, Taiwan
3. Drug Development and Value Creation Research Center, Kaohsiung Medical University, Kaohsiung City, Taiwan
4. Sleep Center, Taipei Veterans General Hospital, Taipei City, Taiwan
5. Department of Psychiatry, Cheng Hsin General Hospital, Taipei City, Taiwan
6. Department of Psychiatry, Faculty of Medicine, National Yang-Ming University, Taipei. Taiwan

*Correspondence Authors

Li-Wei Ko: lwko@nctu.edu.tw

# Supplementary

Table S1. Questionnaire A. This questionnaire was filled pre-bedtime to ensure the participant was in his/her normal state.

| Items | Brief introduction |
| --- | --- |
| 1. Date | Fill out days. |
| 2. I went to bed at: |  |
| 3. I exercised at least 20 minutes in the: |  |
| 4. Approximately 2-3 hours before going to bed, I consumed: |  |
| 5. The degree of you felt sleepy: | Record score  (response scale: 1 “very alter” to 8 ”almost fell asleep” ) |
| 6, How you felt now: |  |
| 7, Were there any difference between today and the usual? | Yes or No (If Yes, describe the different parts) |
| 8. Did you take a nap today? | Yes or No (If Yes, for how long?) |
| 9. Did you feel uncomfortable now? | Yes or No |
| 10. Were you getting ready for bed? | Yes or No |
| 11.Note | Record any other factors that may affect your sleep  (i.e. hours of work shift or periods). |

Table S2. Questionnaire B. This questionnaire was filled after each sleep session to evaluate the participant’s objective sleep quality.

| Items | Brief introduction |
| --- | --- |
| 1. Date | Fill out days. |
| 2. Last night, I fell asleep in: |  |
| 3. I woke up during the night: | Record number of hours |
| 4. I got up of bed this morning at: |  |
| 5. Last night I slept a total of: | Record number of hours |
| 6. My sleep was disturbed by: | List any mental, emotional, physical or environmental factor |
| 7. Was the sleep time as long as your usual sleep routine? |  |
| 8. Was there any difference of sleep state between today and the usual? |  |
| 9. How you felt now? |  |
| 10. How did you wake up? |  |
| 11. Did you remember your dream from last night? |  |
| 12. Did you feel uncomfortable now? |  |
| 13. Sleep quality index  Q1: The level of sleep depth?  Q2: The length of sleep duration?  Q3: The times of sleep disturbance?  Q4: Did you sleep well? | Record score (response scale: 1 to 7)  Q1: 1 “light sleep” to 7 “deep sleep”  Q2: 1 “short time” to 7 “long sleep”  Q3: 1 “less disturbance” to 7 “much disturbance”  Q4: 1 “very bad” to 7 “very good” |

Table S3. The SSS questionnaire scales. A scale was recorded every 2 hours in daytime to evaluate the sleepiness of the participants.

| Degree of Sleepiness | Scale Rating |
| --- | --- |
| Feeling active, vital, alert, or wide awake | 1 |
| Functioning at high levels, but not at peak; able to concentrate | 2 |
| Awake, but relaxed; responsive but not fully alert | 3 |
| Somewhat foggy, let down | 4 |
| Foggy; losing interest in remaining awake; slowed down | 5 |
| Sleepy, woozy, fighting sleep; prefer to lie down | 6 |
| No longer fighting sleep, sleep onset soon; having dream-like thoughts | 7 |
